# Stress during pregnancy: An ecological momentary assessment of stressors among Black and White women with implications for maternal health

**DOI:** 10.1177/17455057221126808

**Published:** 2022-09-23

**Authors:** Serwaa S Omowale, Tiffany L Gary-Webb, Meredith L Wallace, John M Wallace, Mary E Rauktis, Shaun M Eack, Dara D Mendez

**Affiliations:** 1 California Preterm Birth Initiative, University of California San Francisco, San Francisco, CA, USA; 2 Department of Obstetrics, Gynecology & Reproductive Sciences, School of Medicine, University of California San Francisco, San Francisco, CA, USA; 3 School of Social Work, University of Pittsburgh, Pittsburgh, PA, USA; 4 Department of Epidemiology, School of Public Health, University of Pittsburgh, Pittsburgh, PA, USA; 5 Department of Psychiatry, University of Pittsburgh, Pittsburgh, PA, USA; 6 Department of Behavioral and Community Health Sciences, School of Public Health, University of Pittsburgh, Pittsburgh, PA, USA; 7 Division of General Internal Medicine, School of Medicine, University of Pittsburgh, Pittsburgh, PA, USA

**Keywords:** determinants, ecological momentary assessment, health status disparities, maternal health, pregnancy, psychological stress

## Abstract

**Background:**

Stress can lead to adverse physiological and psychological outcomes. Therefore, understanding stress during pregnancy provides insight into racial disparities in maternal health, particularly Black maternal health.

**Objectives:**

This study aimed to describe (1) daily exposure to self-reported stress levels during pregnancy, and (2) sources of stress among participants that identified as Black or White using data collected via ecological momentary assessment.

**Methods:**

We leveraged survey data from the Postpartum Mothers Mobile Study, a prospective longitudinal study using ecological momentary assessment data collection methods to describe patterns of stress during pregnancy. This article is descriptive and documents patterns of self-reported stress levels and sources of stress. Frequencies and percentages of stress responses were computed to describe these patterns.

**Results:**

The sample (*n* = 296) was 27% Black (*n* = 78) and 63% White (*n* = 184). Results were based on at least one measurement of that stress level during pregnancy. A similar number of Black and White participants reported no stress during pregnancy. White (85%–95%) and Black (60%–70%) participants reported low to moderate levels of stress. Black participants (38%) and White participants (35%) reported experiencing high stress. Black and White participants reported similar sources of stress: stress from a partner, too many things to do, a baby or other children, and financial concerns. White participants reported work as a top stressor, and Black participants reported financial issues as a top source of stress.

**Conclusion:**

This study provides insight into daily exposure to stress that has implications for maternal health. We described patterns of self-reported stress and sources of stress among Black and White participants. The daily exposures to stress reported by this sample exist within a context of root causes of structural inequities in education, health care, income, wealth, and housing that must be addressed to achieve maternal health equity.

## Introduction

Psychological stress is the relationship between a person and the environment, as people cognitively appraise the stress as taxing and exceeding the necessary resources to cope.^1^ Stress is unavoidable, and an inadequate response to threatening (i.e. harmful) stimuli in the environment can lead to adverse physiological and psychological outcomes.^1,2^ Stress during pregnancy has been associated with experiences of domestic violence, chronic health problems,^3^ late prenatal care initiation,^4^ sexual and racial discrimination,^5^ financial strain,^6^ food insecurity, job strain, and caring for an ill family member.^7^ Several studies found that stress during pregnancy is associated with adverse birth outcomes, including low birth weight (birth weight less than 2500 g).^8–10^ Furthermore, stress during pregnancy has been linked to several adverse reproductive outcomes, such as bacterial vaginosis, changes in neuroendocrine process (i.e. fight-or-flight response), and cardiovascular disorders (e.g. maternal hypertension).^11,12^ Also, responses to stress are associated with harmful health behaviors such as smoking and illicit drug use.^3^ Therefore, the inability to cope with stress due to lack of resources (e.g. income) and structural barriers (e.g. affordable housing) in society can lead to adverse health outcomes. Hence, understanding exposures to stressors during pregnancy is vital to improving maternal health.

Racial disparities in perinatal health outcomes are a persistent public health issue in the United States. Pregnancy-related deaths among Black women are three times higher (2007–2016) than their White counterparts.^13^ Since psychological stress has been linked to negative reproductive health outcomes, it may explain factors contributing to higher rates of maternal mortality among Black women. A previous study found that Black women experience higher psychosocial stressors (e.g. discrimination) and biomarkers of stress (e.g. corticotropin-releasing hormone, adrenocorticotropic hormone) than White women during pregnancy.^14^ Other studies have found that Black women experienced a threefold increased risk for posttraumatic stress disorder symptoms during pregnancy^15^ and report a higher burden of psychosocial stressors, like racism, than non-Black women.^16^ In addition, Black and White women specify different stressors during pregnancy across sociodemographic characteristics.^8,17^ Thus, understanding exposure to stressors during pregnancy in diverse racial populations is vital to addressing racial disparities in adverse pregnancy outcomes.

The association between stress and racial disparities in pregnancy outcomes is poorly understood. Few studies have found that maternal stress explained Black–White differences in adverse birth outcomes,^18,19^ another study found that stress did not explain these differences.^20^ Also, most studies use cross-sectional study designs that rely on a single exposure to stress or retrospective recall. These study designs are limited in assessing real-time, chronic, or long-term exposure to stress during pregnancy.

### Ecological momentary assessment

This study used ecological momentary assessment (EMA) data collection methods to examine exposure to stress during pregnancy. EMA is a promising data collection method because it captures exposures, experiences, and behaviors as they occur in real-time and in the natural settings of study participants.^21^ Furthermore, capturing real-time data reduces recall bias among study participants and increases ecological validity. This data collection method also reduces interviewer and participant bias that may occur due to the data collection in clinical settings or environments that are unnatural to study participants. EMA data collected via smartphone technology (e.g. mobile application) or other handheld devices can also increase participant access and engagement in studies.^22^

EMA is a feasible data collection method among pregnant populations.^23,24^ Several EMA studies have examined substance use disorders,^25,26^ breastfeeding,^27^ obesity,^28^ and health promotion interventions^29^ in pregnant and postpartum populations. Prior studies using EMA methods have not examined exposure to several stressors in a diverse pregnant population longitudinally, which created the impetus for this study. This study aims to describe (1) daily exposure to self-reported stress levels during pregnancy, and (2) sources of stress among participants that identified as Black or White using data collected via EMA. This descriptive analysis will be helpful for understanding the current state of stressors in Black and White perinatal populations and help inform future hypothesis-testing studies.

## Methods

### Study overview

This study is a secondary analysis using data from the Postpartum Mothers Mobile Study (PMOMS), a prospective longitudinal study examining factors associated with racial disparities in postpartum weight and health during and after pregnancy using EMA and non-EMA data collection methods.^30,31^ PMOMS is ancillary to the GDM^2^ comparative effectiveness trial focused on assessing two testing strategies for gestational diabetes.^32^ A total of 284 participants were recruited from the GDM2 Trial and another 29 were recruited directly from prenatal care clinics (after the GDM2 Trial ended) in a single woman’s hospital in southwestern Pennsylvania. Recruitment began in December 2017 and ended in March 2020 due to the pandemic.

Study participants were recruited during the second and third trimester of pregnancy (18–28 weeks of gestation). Participants were followed from 18–24 weeks’ gestation through 1 year postpartum for an average of 15 months. Potential participants with chronic health conditions (e.g. preexisting type 1 or 2 diabetes mellitus) and several health comorbidities were excluded from study participation. The full exclusion criteria are published elsewhere.^32^ EMA data collected during pregnancy January 2018 through June 2020 were included in this analysis. All study participants delivered by June 2020. The study population included a diverse racial/ethnic and sociodemographic sample of participants aged 18–45 years old with a singleton pregnancy. All participants provided written consent before enrolling in the study. Once participants consented to the study, they completed baseline surveys, received a smartphone and smart scale, and received instructions on how to respond to EMA surveys using web-based technology.^30,32^

The University of Pittsburgh Human Research Protection Office approved all study protocols beginning in October 2017 (IRB #PRO16100117).

### Measurement

#### Ecological momentary assessment

EMA survey data are collected using smartphone technology through a web-based mobile application. Participants used smartphones issued by the study or personal smartphones to complete surveys. Data were collected via EMA using signal-contingent (i.e. random times during the day) and time-contingent (i.e. fixed times) prompts on participants’ smartphones. Each day, participants were prompted to complete a series of EMA items on their smartphones at the beginning of the day (BOD) and at the end of the day (EOD) set by participant preferences (e.g. at 8:00 a.m. daily).^30^ For this analysis, data collected via signal-contingent prompts (i.e. random surveys) sent during a 12-h waking period (i.e. the hours between the morning (BOD) and evening prompts (EOD) that included questions about mood, stress, self-efficacy, microaggressions, race, and gender discrimination were used. EMA prompts for the “random survey” were delivered zero to three times per day to achieve a target of one mean random assessment over a 7-day period.^30^ Further explanation about the design and sampling frame for EMA surveys are published elsewhere.^30^ Overall, the completion rate among the PMOMS cohort for signal-contingent random surveys was 75.5% during pregnancy.

#### Stress variables

Self-reported stress levels were assessed via random EMA surveys using questions adapted from the Perceived Stress Scale (PSS).^33^ The original PSS is a 14-item five-point Likert-type scale that measures the impact of life events and perceived stress within the last month with response options ranging from never to very often. The scale has been determined to have adequate internal and test–retest reliability and is a standard measure of self-reported perceived stress used in over 100 studies.^33^ The scale has been adapted in other studies into 10-item and 4-item versions.^34^ Prior studies have used this measure to assess maternal stress.^5,35–39^ We used one item adapted from the PSS to assess perceived stress in real-time using survey data collected via smartphones. Other EMA studies have used a single question to assess perceived stress in diverse populations.^40–42^ In this study, we asked participants to “rate if you are feeling nervous or stressed right now,” with a rating scale ranging from 0 (not at all) to 4 (a lot). If participants selected a stress rating of 1 or greater, they were prompted to indicate the sources of stress. Participants could select one or more of the following: work-related, baby or other children, partner or spouse, other family member or friend, financial issues, housing issues, too many things to do at once, and other (specify). Written responses specifying other sources of stress were not included in this analysis.

#### Analytic sample

All participants who responded to the primary stress item at least once via the random survey during pregnancy were included in this analysis. Therefore, participants without at least one observation were excluded from the analysis and determined not to have the exposure of interest. The full study sample consisted of 313 participants. One participant voluntarily withdrew from the study and never completed any random surveys. Of the remaining 312 participants, 305 participants had a response to the stress item on the random survey. Of those participants, nine participants were excluded because they only responded to the stress item during the postpartum period. The analytic sample of participants that completed responses to the stress item on the random survey during pregnancy is 296. Two of the 296 participants voluntarily withdrew from the study during pregnancy. A subset of the analytic sample was used for stratified analyses for Black (*n* = 78) and White (*n* = 184) participants. Participants that responded to the primary stress item (rating 0–4) on the random survey and only selected 0 were not prompted to select any sources of stress. Only 275 participants contributed data to sources of stress. A subset of Black (*n* = 69) and White (*n* = 179) participants that contributed data to the sources-of-stress variables (e.g. work-related, baby) were used for stratified analyses.

#### Descriptive methods

The analysis for this study is descriptive and explores patterns of self-reported stress levels and sources of stress during pregnancy (second and third trimester) using data from EMA surveys. Frequencies and percentages of EMA stress responses were computed to describe these patterns. The frequencies and percentages of observations that contributed to a specific stress level rating (ranging from 0 to 4) were computed to describe the patterns of stress levels. Next, the frequencies and percentages of participants that ever selected a particular response to the stress item and sources of stress (e.g. work-related, baby) at any given time during pregnancy were computed. Frequencies and percentages were also computed to describe the demographics of the study sample. All analyses were stratified by participants that identified as Black and White, and by educational level. These analyses were conducted using Stata/SE, version 16.1 (StataCorp, College Station, Texas).

## Results

### Demographic characteristics

Table 1 displays baseline self-reported sociodemographic information for the analytical sample. Two participants out of the sample of 296 had missing self-reported race data. Two participants who identified as White, non/Hispanic withdrew voluntarily from the study during pregnancy. Both participants were included in this analysis; one of the participants contributed 8 responses to the stress item, and the other contributed 46 responses. The study sample was 27% Black (*n* = 78) and 63% White (*n* = 184). Out of Black participants, 16.67% were married (*n* = 13), 52.56% were employed full or part time (*n* = 41), 17.95% had a 4-year college degree or higher (*n* = 14), and 7.69% had an annual household income of US$51,000 or greater (*n* = 6). Out of White participants, 73.37% were married (*n* = 135), 77.72% were employed full or part time (*n* = 143), 73.28% had a 4-year college degree or higher (*n* = 133), and 71.20% had an annual household income of US$51,000 or greater (*n* = 131). The average number of random surveys completed by the overall sample was 50.7 (range = 1–102). Black participants completed an average of 41.4 random surveys (range = 2–90), and White participants completed an average of 54.6 random surveys (range = 1–102) during pregnancy.

**Table 1.** Demographic characteristics for study population (*N* = 296), stratified by Black (*n* = 78) and White (*n* = 184) participants.

Characteristic	All participants	Black participants	White participants
	*n* (%)	*n* (%)	*n* (%)
Race and ethnicity			
Asian	12 (4.08)		
Native Hawaiian/Other Pacific Islanders	1 (0.34)		
Black/African American	78 (26.53)		
White	184 (62.59)		
Multiracial	9 (3.06)		
Other^a^	10 (3.40)		
Missing	2		
Hispanic (yes)^b^	18 (6.08)		
Student status (yes)	31 (10.47)	10 (12.82)	13 (7.07)
Age (years)			
18–24	40 (13.51)	21 (26.92)	14 (7.61)
25–29	96 (32.43)	32 (41.03)	52 (28.26)
30–34	110 (37.16)	22 (28.21)	81 (44.02)
35+	50 (16.89)	3 (3.85)	37 (20.11)
Employment status			
Working part-time	44(14.86)	10 (12.82)	27 (14.67)
Working full-time	163 (55.07)	31 (39.74)	116 (63.04)
Receiving disability benefits	5 (1.69)	4 (5.13)	0
Unemployed or not working	84 (28.38)	33 (42.31)	41 (22.28)
Educational level			
Less than high school	15 (5.07)	8 (10.26)	6 (3.26)
High school diploma or GED^c^	51 (17.23)	32 (41.03)	13 (7.07)
Some college or vocational degree	60 (20.27)	24 (30.77)	32 (17.39)
College degree	79 (26.69)	14 (17.95)	58 (31.52)
Masters degree	57 (19.26)	0	50 (27.17)
Doctoral, law, or medical degree, or higher	34 (11.49)	0	25 (13.59)
Household income (annual in US$)			
Less than 20,000	71 (23.99)	43 (55.13)	21 (11.41)
21,000–30,000	35 (11.82)	19 (24.36)	14 (7.61)
31,000–40,000	16 (5.41)	5 (6.41)	7 (3.80)
41,000–50,000	18 (6.08)	5 (6.41)	11(5.98)
51,000–60,000	22 (7.43)	4 (5.13)	16 (8.70)
61,000–70,000	12 (4.05)	0	11 (5.98)
71,000–80,000	19 (6.42)	0	13 (7.07)
81,000 or greater	103 (34.80)	2 (2.56)	91 (49.46)
Marital status			
Single/never married	110 (37.16)	60 (76.92)	43 (23.37)
Separated or divorced	10 (3.38)	4 (5.13)	6 (3.26)
Married	175 (59.12)	13 (16.67)	135 (73.37)
Other	1 (0.34)	1 (1.28)	0

a Includes individuals who identify as Middle Eastern or South American.

b Participants selected from racial categories and/or Hispanic or non-Hispanic.

c General educational development certification.

### Stress outcomes

Table 2 includes the frequencies and percentages of observations that contributed to a specific stress level rating (ranging from 0 to 4). Also, the frequencies and percentages of participants that ever selected a particular response are presented in the table. Responses to the stress levels are presented in the table by both observations and participants to provide an overview of the responses included in the total sample (*n* = 296) collected via EMA over the second and third trimesters of pregnancy. Most participants (96%) reported they experienced no stress at some point during pregnancy. Overall, most participants (76%–86%) reported they experienced low to moderate stress (rating 2 or 1) during pregnancy with the highest number of observations (11–20%) contributed to this level of stress. However, 35.14% (*n* *=* 104) of participants reported experiencing the highest stress level (rating 4) at some point during pregnancy.

**Table 2.** Frequencies and percentages of observations for perceived stress: study population (*N* = 296).

	Observation (*n*)	Observation (%)	Participants who selected response^a^ (*n*)	Participants who selected response^b^ (%)
Stress level 0	8906	59.36	285	96.28
Stress level 1	3117	20.77	255	86.15
Stress level 2	1675	11.16	227	76.69
Stress level 3	853	5.69	174	58.78
Stress level 4	453	3.02	104	35.14
Total	15,004	100		

a The number of participants that ever selected a specific response.

b The percentage of participants out of the total sample (*N* = 296) who ever selected a specific response.

Table 3 includes frequencies and percentages of participants that ever selected a specific level of stress rating (ranging from 0 to 4) stratified by participants that identified as Black or White. The stress results were based on at least one measurement of that stress level during pregnancy. A similar number of Black and White participants reported experiencing no stress during pregnancy. Overall, White participants (85%–95%) reported low to moderate levels of stress (rating 2 or 1) and (60%–70%) of Black participants reported low to moderate stress. A lower number of participants reported ever experiencing high stress during pregnancy. Black participants (38%) and White participants (35%) indicated high stress (rating 4) during pregnancy.

**Table 3.** Frequencies and percentages of reported perceived stress, stratified by Black and White participants.

	Black participants (*n* = 78)		White participants (*n* = 184)	
	Participants who selected response^a^ (*n*)	Participants who selected response^b^ (%)	Participants who selected response^a^ (*n*)	Participants who selected response^b^ (%)
Stress level 0	75	96.15	177	96.20
Stress level 1	55	70.51	175	95.11
Stress level 2	47	60.26	157	85.33
Stress level 3	39	50.00	114	61.96
Stress level 4	30	38.46	64	34.78

a The number of participants who ever selected a specific response.

b The percentage of participants out of the total sample (78 Black participants and 184 White participants) who ever selected a specific response.

### Sources of stress

Table 4 summarizes the frequencies and percentages of participants that ever selected a specific stressor (e.g. work, baby) stratified by participants that identified as Black or White during the second and/or third trimester of pregnancy. Only participants who responded with a 1 or higher stress level rating (ranging from 0 to 4) to the question asking them to “rate if you are feeling nervous or stressed right now” contributed responses to the sources-of-stress items, resulting in a total of 69 Black and 179 White participants for these analyses. Participants had the option to select several items they considered sources of stress. Only the “yes” responses are included in the table.

**Table 4.** Frequencies and percentages of sources of stress, stratified by Black and White participants.

	Black participants (*n* = 69)		White participants (*n* = 179)	
	Participants who selected response^a^ (*n*)	Participants who selected response^b^ (%)	Participants who selected response^a^ (*n*)	Participants who selected response^b^ (%)
Stress: work	31	44.93	136	75.98
Stress: baby/other children	50	72.46	144	80.45
Stress: partner	36	52.17	103	57.54
Stress: family	18	26.09	100	55.87
Stress: financial	42	60.87	92	51.40
Stress: housing	34	49.28	55	30.73
Stress: too many things to do at once	45	65.22	153	85.47
Stress: other	28	40.58	121	67.60

a The number of participants who ever selected a specific response.

b The percentage of participants out of the total sample who ever selected a specific response.

Black and White participants reported some similar sources of stress with 50% or more selecting a partner, too many things to do, baby or other children, and financial concerns during pregnancy. However, outside of these commonalities, Black and White participants’ responses to sources of stress differed. Black participants indicated that family (26%; *n* = 18) was a low source of stress during pregnancy, while White participants reported housing (31%; *n* = 55) as a low stressor. For White participants, a top source of stress was work-related (76%; *n* = 136). Nearly 50% of Black participants (*n* = 34) reported housing as a stressor during pregnancy.

To further explore stress during pregnancy across sociodemographic factors, we assessed sources of stress by educational level for participants that identified as Black or White. Table 5 summarizes the frequencies and percentages of participants who selected a specific stressor during pregnancy by educational level. Our sample included 14 Black and 131 White participants who reported attaining at least a bachelor’s degree. More White participants indicated they held graduate degrees compared to Black participants. Given the limited sample size in higher levels of education, this analysis does not compare racial groups.

**Table 5.** Frequencies and percentages of sources of stress, stratified by Black and White participants educational levels.

	Black participants who selected response^a^ *n* (%)		White participants who selected response^a^ *n* (%)	
	Less than college education (*n* = 55)	College education (*n* = 14)	Less than college education (*n* = 48)	College education (*n* = 131)
Stress: work	22 (40.00)	9 (64.29)	30 (62.50)	106 (80.92)
Stress: baby/other children	39 (70.91)	11 (78.57)	43 (89.58)	101 (77.10)
Stress: partner	29 (52.73)	7 (50.00)	32 (66.67)	71 (54.20)
Stress: family	15 (27.27)	3 (21.43)	36 (75.00)	64 (48.85)
Stress: financial	34 (61.82)	8 (57.14)	37 (77.08)	55 (41.98)
Stress: housing	26 (47.27)	8 (57.14)	22 (45.83)	33 (25.19)
Stress: too many things to do at once	37 (67.27)	8 (57.14)	42 (87.50)	111 (84.73)
Stress: other	21 (38.18)	7 (50.00)	35 (72.92)	86 (65.65)

a The number and percentage of participants who ever selected a specific response.

Among Black and White participants, there are some similar sources of stress to note across all groups of participants regardless of education. At least 50% or more of the participants selected a partner, too many things to do, and baby or other children as a stressor during pregnancy, which was similar to the results without stratification by educational level.

White participants with college degrees (*n* = 106) reported that work-related stress (81%) was the primary source of stress during pregnancy. Both groups of White participants stated similar top three sources of stress except for financial issues. The 48 White participants without college degrees reported financial issues (77%; *n* = 37) contributed to stress during pregnancy. For both groups of White participants, housing was the lowest source of stress during pregnancy. However, a higher percentage of White participants without college degrees (46%; *n* = 22) indicated housing as a stressor and only 25% (*n* = 33) of college-educated White participants indicated this was a stressor.

Over 50% of college-educated Black participants (*n* = 14) and those without college degrees (*n* = 55) indicated financial issues as a source of stress during pregnancy. The same percentage of Black participants with college degrees reported financial and housing issues (57%; *n* = 8). It is also important to note that over half of college-educated Black participants indicated work as the second highest source of stress during pregnancy (64%; *n* = 9) while only (40%; *n* = 22) without college education. For both groups of Black participants, family was a low source of stress during pregnancy.

## Discussion

In this study, we found that most participants reported a low score (rating 1) for stress during pregnancy based on the Cohen PSS item of stress. We found similar patterns of self-reported stress levels among Black and White participants from responses to the stress item (rating 0–4) during the second and/or third trimester of pregnancy. Most Black and White participants reported low to moderate levels (rating 1 or 2) of stress during pregnancy. Overall, a lower and similar number of Black and White participants reported experiences of high stress (rating 4) during pregnancy.

We also found that Black and White participants shared similar sources of stress during pregnancy, with over 50% or more selecting a partner, too many things to do, baby or other children, and financial concerns during pregnancy. However, there are some low and high stressors outside of these commonalities. About 76% of White participants indicated that work was a top source of stress during pregnancy. In addition, nearly 50% of Black participants indicated that housing was a source of stress. The low stressor for Black participants was family, while housing was low for White participants. About 41% of Black and 68% of White participants indicated other sources of stress during pregnancy outside of the list of potential stressors. Participants had the option to specify these additional stressors by writing in responses, but they were not required to write in responses. We did not analyze these data but would like to highlight some of the written responses among the 296 participants. Most written responses were related to concerns about pregnancy and delivery (e.g. C-section, induction, the physical discomfort of pregnancy). Examples of responses were “beginning induction today,” “birth coming,” “c-section approaching,” “worried about my c-section.” Also, participants expressed concerns about delivery during the COVID-19 pandemic (e.g. “upcoming birth during pandemic”) and generally early on during the pandemic; particularly among the 21 participants who were still being followed during pregnancy between March and June 2020.

To get a nuanced understanding of patterns of stressors in our study sample, we stratified participants by race (Black and White participants) and educational level. Our study sample had a smaller number of college-educated Black participants (*n* = 14) than White participants (*n* = 131). However, some important sources of stress should be noted across the four groups of participants by educational level. At least 50% of all participants, regardless of their educational level indicated a partner, too many things to do, and baby or other children as a stressor during pregnancy. Among college-educated White participants, 81% indicated that work was a source of stress. White participants without a college degree (77%) specified that finances were a concern and only 42% of college-educated White participants indicated this was a concern. Black participants with college education reported a similar pattern of financial stress as Black participants without college degrees.

Our findings show some similar patterns of stress levels and sources of stress during pregnancy among Black and White participants. It is important to note that Black and White participants reported similar experiences of high stress during pregnancy. In addition, a higher number of White participants reported low to moderate stress than Black participants.

The stress patterns observed among Black and White participants in our study may be explained by differences in coping responses. Stress theory identifies two cognitive processes called appraisals that mediate stress responses.^2^ The primary appraisal is a cognitive appraisal in which a person will evaluate whether a stressful encounter is harmful or beneficial. The secondary appraisal is a coping response based on the initial cognitive evaluation of a stressful encounter.^1,43^

The weathering hypothesis suggests that Black women experience a lifetime of accumulated stress and must over-cope with these stressors.^44,45^ Also, several studies have found that racial discrimination is a stressor for Black women.^46–50^ Black women’s appraisal of what is stressful may be more indicative of long-term exposure to stress.^51,52^ Therefore, Black women may appraise daily stressors as less threatening or learn to cope with stress due to ongoing or high exposure to stress. However, coping with stress does not mean stress has no impact on health outcomes. Weathering has been assessed using allostatic load scores (“price the tissue of organs pay for an overactive or inefficiently managed allostatic response”).^53^ Moreover, the biological impact (“cumulative wear and tear”) of stress on the body and health. Several studies found that Black women have higher allostatic load scores than White women.^54–57^ Other studies found an age-related increased risk for adverse birth outcomes among Black women.^58–60^ These studies suggest that long-term and chronic stress is associated with adverse pregnancy and health outcomes among Black women.

Black and White participants reported similar sources of stress such as a partner, too many things to do, baby or other children, and financial concerns during pregnancy. However, some differences should be highlighted. The majority of White participants in our study were highly educated with graduate degrees and worked full-time at the study baseline. Among White participants, work-related stress was the primary source of stress during pregnancy. White participants in this study may have experienced more work stress because they were employed at higher rates and most likely in professional positions due to graduate education. Employed women in higher demanding professional roles may experience work–family conflict, which involves conflicting roles between work and family domains.^61^ Several studies have found that challenges in career management among women are related to work–family conflict.^62,63^ Lower employment among Black participants may have contributed to lower reports of work-related stress. Most of the Black participants in this study’s sample had less than a college education, affecting their employment opportunities. Hence, lower employment and household incomes of Black participants potentially contributed to financial insecurity and reports of housing issues as sources of stress. Income and resources to address overwhelming daily tasks, current pregnancy concerns, and caregiver obligations may differ for Black and White participants.

Economic stability is a key social determinant of health, which influences health outcomes.^64,65^ Responses to financial and housing issues indicate economic insecurity and were primary sources of maternal stress among Black participants in our study. Understanding maternal stress by educational levels is important since Black women with college education are five times as likely to die from pregnancy-related deaths than their White counterparts, according to the most recent national data (2007–2016) stratified by race/ethnicity and educational levels.^13^ This study found that Black women reported financial and housing issues, which may result from economic insecurity. In the maternal and child health literature, it is well established that lower socioeconomic status is a risk factor for adverse birth outcomes.^66–69^ In addition, several studies found that pregnant Black women experience racial bias in the health-care system.^70–72^ Moreover, structural racism^73,74^; discrimination through housing, education, employment, health care, and criminalization systems influence ongoing social inequities contributing to racial disparities in adverse pregnancy outcomes.

Participants for this study were recruited from a maternity hospital in Pittsburgh, PA (Allegheny County), and surrounding counties. Most of the deliveries (70%) at the hospital between 2019 and 2020 were from residents of Allegheny County. Pittsburgh, PA is the largest city in the county and in southwestern Pennsylvania. In 2019–2020, about 20% of the patients that delivered at the hospital were Black and 70% were White. According to the Census Bureau, an estimated 14% of Allegheny County residents were Black, and 79% were White in 2021.^75^ Thus, our study sample was similar to patients that identified as Black or White in the hospital population that delivered a baby between 2019 and 2020. Although participants in our study may have lived outside of Pittsburgh and Allegheny County, it is vital to note social inequities across race and gender in the city of Pittsburgh. In Pittsburgh, Black women are twice as likely to live in poverty as compared to White women, and one-third live below the federal poverty line.^76^ Moreover, the social inequities in the local population provide some insight into the different social demographic characteristics of Black and White participants in our study sample.

Although most study participants delivered before 2020, the authors would be remiss not to mention the impact of the COVID-19 pandemic on participants enrolled in the study during this period. One of our prior analysis found an increase in stress during emergency declaration/stay-at-home orders in the United States among pregnant and postpartum participants that were still enrolled in PMOMS at the onset of the COVID-19 pandemic.^40^ In addition, stress among pregnant and postpartum populations during the pandemic is associated with fear of virus infection,^77–79^ loss of employment and changes to work environments,^80^ uncertainty related to delivery plans and prenatal care,^78,79^ and limited support due to hospital restrictions.^77^ Other potential stressors are limited childcare options,^81,82^ increased gendered role responsibilities, such as caring for family members or children; and reduction in working hours or productivity to attend to family obligations.^83–85^ In addition, the differential impact of COVID-19 infections^86,87^ and death^88^ among Black Americans potentially influenced racial differences in reports of stress.

### Limitations

The current findings should be considered in the context of some limitations. First, participants may or may not complete random surveys daily since they are signal-contingent (i.e. random) prompts unlike time-contingent (i.e. fixed times) set to participant preferences. However, 75.5% of the random surveys were completed by participants in our sample during pregnancy. Second, participant responses to the stress measurements time points and frequency of data collection varied over the study period. Moreover, our analysis does not account for within and between cluster correlations at the participant level. This study does provide descriptive analyses using percentages to indicate the number of participants that selected a stress rating or sources of stress during pregnancy.

Since this study was a secondary analysis, a calculation for the sample size was not performed for this study. We did not conduct significance testing to determine a statistical difference in stress levels or sources of stress by race. Therefore, comparisons by race should not be viewed as significantly different. The goal of this study was to describe stress patterns during pregnancy among Black and White participants as measured by EMA, a novel data collection method. These descriptive analyses can be used to generate future informed hypotheses related to stress in real-time and racial disparities during pregnancy.

### Strengths

This study has several strengths to note. First, the study sample was diverse across sociodemographic characteristics, which allowed for the examination of differences in experiences of stress during pregnancy among persons that identify as Black or White. Second, survey responses collected via smartphone technology provided insight into participants’ experiences in real time and in their natural environments. Moreover, EMA methods provided data on repeated exposure to stress and stressors during pregnancy at the participant level. Also, the random sampling design provided a representative sample of participants’ responses during their time in the study. This study extends our understanding of the current state of stressors in Black and White perinatal populations using data collected in real time via EMA methods.

In summary, EMA survey methods allowed for the collection of survey data over several time points in real time and as participants experienced different stress levels or stressors throughout their pregnancy. Our analysis summarized these stress patterns and assessed differences by race. Unlike cross-sectional studies that usually collect survey data only at baseline and traditional longitudinal studies that have fewer points of data collection, this study collected surveys at several time points during pregnancy. Moreover, this study captures survey data regarding experiences of stress over time, which would be missed or underreported using traditional longitudinal or cross-sectional study designs.

## Conclusion

This study explored self-reported stress and several sources of stress during pregnancy using EMA data collection methods via smartphone technology. To our knowledge, this is the first EMA study examining self-reported stress and stressors during the second and third trimesters of pregnancy in a racially diverse sample. Study findings suggest some similar patterns of stress regardless of race; however, there were different sources of stress among Black and White participants. In addition, participant responses to the stress items suggest that EMA data collection is a feasible method to examine stress and sources of stress in perinatal populations.

If these findings are confirmed in future hypothesis testing studies, they will have implications for policy action and a public health response to address maternal health and birth equity. Black participants reported financial and housing issues as sources of stress during pregnancy, which are key indicators of economic instability. Policies actions are needed to address the persistent inequities that create the social and physical environments and quality of life of racialized groups in the United States. While prioritizing and extending access to Medicaid medical assistance programs (during pregnancy and postpartum), affordable housing, food benefits, doula services (i.e. birth support workers), home visiting programs, and targeted policy and interventions to improve perinatal medical protocols would improve maternal health. The root causes of structural inequities in education, health care, income, wealth, and housing influenced by the legacy of enslaving African people, Jim Crow segregation, and ongoing racial and gendered discrimination against Black Americans must be addressed as a public health intervention to improve maternal health for Black families and communities.

## Supplemental Material

sj-pdf-1-whe-10.1177_17455057221126808 – Supplemental material for Stress during pregnancy: An ecological momentary assessment of stressors among Black and White women with implications for maternal health Click here for additional data file. Supplemental material, sj-pdf-1-whe-10.1177_17455057221126808 for Stress during pregnancy: An ecological momentary assessment of stressors among Black and White women with implications for maternal health by Serwaa S Omowale, Tiffany L Gary-Webb, Meredith L Wallace, John M Wallace, Mary E Rauktis, Shaun M Eack and Dara D Mendez in Women’s Health

## Declarations

**Ethics approval and consent to participate:** The University of Pittsburgh Human Research Protection Office approved all study protocols beginning in October 2017 (IRB #PRO16100117). All eligible participants provided verbal and written consent to participate in the Postpartum Mothers Mobile Study (PMOMS).

**Consent for publication:** The consent to participate in the study includes consent to publish study findings. All data included in this study are de-identified.

**Availability of data and materials:** Study data and materials are available upon request from the corresponding author, DDM and approval of The University of Pittsburgh Human Research Protection Office.
