# Supplementary material for: Stress during pregnancy: An ecological momentary assessment of stressors among Black and White women with implications for maternal health
Source: Womens Health (Lond). 2022 Sep 23;18:17455057221126808. doi: 10.1177/17455057221126808 (PMC9510975; doi:10.1177/17455057221126808)
Supplement: sj-pdf-1-whe-10.1177_17455057221126808 – Supplemental material for Stress during pregnancy: An ecological momentary assessment of stressors among Black and White women with implications for maternal health [file sj-pdf-1-whe-10.1177_17455057221126808.pdf]

Postpartum Mothers Mobile Study EMA Stress Measures

Stress-EMA Variables (RAND, During Pregnancy and Postpartum)

Variable	Question	Response
**STRESS**	Rate if you are feeling nervous or "stressed" right now?	Rate on a scale of 0-4:<br>[0=not at all; 4=a lot]
**STRESSWHY**	(If rating is **1** or higher) Describe the source of your stress.	(check all that apply)<br>1=Work-related **STRESSWORK**<br>2=Baby or other children **STRESSBABY**<br>3=Partner/spouse **STRESSPARTNER**<br>4=Other family member/friend **STRESSFAMILY**<br>5=Financial issues **STRESSMONEY**<br>6=Housing issues **STRESSHOUSE**<br>7=Too many things to do at once **STRESSTODO**<br>8=Other (please specify) **STRESSOTH**, **STRESSOTHS**
